# Enhanced Microencapsulation of C-Phycocyanin from *Arthrospira* by Freeze-Drying with Different Wall Materials

**DOI:** 10.17113/ftb.58.04.20.6622

**Published:** 2020-12

**Authors:** Wanida Pan-utai, Siriluck Iamtham

**Affiliations:** 1 Institute of Food Research and Product Development, Kasetsart University, Chatuchak, 10900 Bangkok, Thailand; 2 Department of Science, Faculty of Liberal Arts and Science, Kasetsart University, Kamphaeng Saen Campus, 73140 Nakhon Pathom, Thailand; 3 Center for Agricultural Biotechnology, Kasetsart University, Kamphaeng Saen Campus, 73140 Nakhon Pathom, Thailand; 4 Center of Excellence on Agricultural Biotechnology: (AG-BIO/PERDO-CHE), 10900 Bangkok, Thailand; 5 Center for Advanced Studies in Tropical Natural Resource, NRU-KU, Kasetsart University, Chatuchak, 10900 Bangkok, Thailand; 6 Research Unit of Orchid Tissue Culture, Kasetsart University, Kamphaeng Saen Campus, 73140 Nakhon Pathom, Thailand

**Keywords:** C-phycocyanin, *Arthrospira*, microencapsulation, freeze-drying, antioxidant properties

## Abstract

**Research background:**

C-phycocyanin (C-PC), a water-soluble blue pigment, was extracted from microalgae *Arthrospira* sp. C-PC could be a good substitute for synthetic pigments with high antioxidant activity. However, C-PC is unstable due to sensitivity to temperature, light, pH and oxygen; therefore, applications of C-PC in food and other products are limited. Microencapsulation of C-PC using freeze-drying is a solution to this problem and is considered a suitable method for drying the heat-sensitive pigment.

**Experimental approach:**

C-phycocyanin was extracted from *Arthrospira platensis*. C-PC microcapsules were modified by freeze-drying, with maltodextrin and gum Arabic used as microencapsulation wall materials at different fractions from 0 to 100%. The physical properties including moisture content and water activity, solubility, hygroscopicity, bulk density, colour appearance, particle morphology and size distribution of the produced powders were evaluated. Thermal stability and antioxidant activity of freeze-dried microencapsulated C-PC powders were also assessed.

**Results and conclusions:**

Freeze-dried microencapsulated C-PC powders with maltodextrin and gum Arabic as wall materials gave high encapsulation efficiency of around 99%. At higher gum Arabic mass fraction, moisture content decreased and water activity improved. Maltodextrin gave higher solubility of C-PC powders whereas gum Arabic led to a similar colour of C-PC to those without microencapsulation. Freeze-dried microencapsulated C-PC powders were composed of different sized microparticles regardless of the combination of wall materials with amorphous glassy shapes. Thermal stability of encapsulated C-PC increased and also showed high antioxidant properties.

**Novelty and scientific contribution:**

This study demonstrates that the freeze-dried microencapsulated C-PC powders have pigment stability with antioxidant properties and are resistant to high temperatures. Therefore, they may have a potential for the development of microencapsulated C-PC as a functional ingredient with improved colour and bioactive properties. Such a product can be applied in food, cosmetic, biotechnology and nutraceutical industries.

## INTRODUCTION

Colour is one of the most important attributes in the food industry and it greatly influences product acceptability by consumers (*1*). Blue colour is rare in nature and bright blue colour of food is often artificial (*2*). Increasing consumer health awareness has highlighted toxicity levels of synthetic colourants used in food (*3*). Seeking naturally derived blue-shaded colourants to replace artificial additives has recently become a major challenge for the food, pharmaceutical and cosmetic industries (*2*, *4*).

The natural blue colour of C-phycocyanin (C-PC) is produced by the photoautotrophic cyanobacteria *Arthrospira platensis* (namely *Spirulina*). *Arthrospira* is considered a nontoxic, non-carcinogenic natural blue colourant for food and cosmetic applications (*5*). Moreover, the US Food and Drug Administration (FDA) classified *Arthrospira* extract as a colour additive exempt from certification and approved its use for confectionery (including sweets and chewing gum), frostings, ice cream and frozen desserts, dessert coatings and topping, beverage mixers and powders, yoghurts, custards, puddings, cottage cheese, gelatine, breadcrumbs and ready-to-eat cereals. In the European Union, *Arthrospira* extract is classified as colouring foodstuff (*2*). Nowadays, food manufacturers are actively looking for natural additives (*6*). Protein content of *Arthrospira* ranges from 50 to 70% dry mass with C-phycocyanin phycobiliprotein as the major source (*7*).

C-phycocyanin is a water-soluble light-harvesting pigment-protein complex and offers many applications as a natural colourant for food and cosmetics (*8*). Interest in natural sources of C-PC has been growing because they may promote human health. Previous reports suggested various C-PC properties as antioxidant, anticancer, anti-inflammatory and other bioactivities which decrease the level of oxidation, thereby promoting healthy cells with potential therapeutic applications (*9*). C-PC is already used as a colourant; however, the natural blue colour is unstable in aqueous solutions.

Microencapsulation is defined as a process of packaging solids, liquids, gases or sensitive ingredients, called core materials, in coating or wall materials to form capsules that are micrometres to millimetres in size based on a drying technique (*10*). The wall materials protect the sensitive ingredients from external influences, control their release and sometimes convert liquids into powders, which are easier to handle (*11*). Various kinds of microencapsulation techniques such as emulsification, coacervation, spray drying, spray cooling, freeze-drying, fluid bed coating and extrusion have been developed (*12*). C-PC encapsulation was studied using alginate and chitosan following the extrusion method (*13*, *14*). However, the final product of C-PC encapsulation is required as a dry ingredient for ease of manufacture or consumption.

Among microencapsulation techniques, freeze-drying, or lyophilisation, is a process used to dehydrate heat-sensitive ingredients (*15*). The drying technique and material used as coating usually affect the retention capacity of ingredients within the matrix (*16*).

Using different wall materials resulted in different chemical properties of the microencapsulated powders such as moisture content, water activity, hygroscopicity and shelf life, depending on the structure and characteristics of each wall material (*17*). Water plays a vital role as a major component of food products and influences food safety, stability, quality and physical properties (*18*). The solubility parameter is associated with reconstitution of powder, while hygroscopicity is essential for powder stability and storage (*19*). The use of natural colourants is an important factor for dried products (*20*). Colour is defined in terms of luminosity (L*), from red to green (a*) and from yellow to blue (b*). Differential scanning calorimetry (DSC) and thermogravimetric analysis (TGA) are important tools in determining the thermal behaviour of microencapsulated natural colourants and their potential use in food (*21*).

It is important to consider the type of wall material used in the microencapsulation process because this may influence the encapsulation efficiency and stability of the capsules (*22*). Maltodextrins (MD) with different molecular masses are products of hydrolysed starch and are commonly used as wall materials for microencapsulation, especially those with dextrose equivalent (DE) between 10 and 20. MD offers advantages due to its low cost, high water solubility, neutral aroma and taste, low viscosity at high solid concentration and low sugar content (*23*, *24*). Moreover, gum Arabic (GA) is a heteropolysaccharide with unique properties of emulsification, low cost and high solubility (*25*). Combination of different types of wall materials can increase encapsulation efficiency (*26*).

Selection of suitable wall materials is important to enhance the efficiency and properties of C-PC microcapsules as a coloured bioactive compound in food applications. Here, C-PC extracted from *A. platensis* was selected to produce microencapsulated powders by freeze-drying using different fractions of maltodextrin 10 DE and gum Arabic as wall materials. Physical properties and thermal analysis of the C-PC microcapsules were evaluated.

## MATERIALS AND METHODS

### Arthrospira microalgal preparation

*Arthrospira platensis* IFRPD 1182 microalgae were sourced from the Institute of Food Research and Product Development (IFRPD), Kasetsart University, Thailand. *Arthrospira* biomass production was generated in 500-litre open raceway ponds (IFRPD, Kasetsart University, Thailand) of working volume of 200 L in Zarrouk medium (*27*). The biomass was grown to exponential phase, harvested by nylon filtration and then cleaned with tap water to remove residual culture medium. *A. platensis* biomass was dried in a hot air oven (model UT6760; Thermo Scientific Heraeus Heating and Drying Ovens, Thermo Fisher Scientific Inc., Thermo Scientific, Dreieich, Germany) at 60 °C for 4-6 h and then milled to 0.5 mm particle size.

### Extraction of C-phycocyanin

C-phycocyanin (C-PC) was extracted from *Arthrospira* oven-dried biomass suspended in distilled water at a concentration of 0.06 g/mL and incubated under a controlled temperature of 25 °C for 24 h in the dark. The suspension was then centrifuged at 22 000×*g* for 30 min (Sorvall RC6 Plus superspeed centrifuge; Thermo Fisher Scientific Inc., Thermo Scientific) at 25 °C and C-PC was concentrated using a vacuum evaporator (R215; Buchi Ltd., Flawil, Switzerland) to reduce it to 1/3 of the initial volume and then stored in the dark at 4 °C until further experiments.

### Microencapsulation procedure

Wall materials including maltodextrin (MD) 10 DE (GB/T20884, food-grade powder; Thai Food and Chemical Co., Bangkok, Thailand) and gum Arabic (GA) (KB-120, food-grade powder; MT Instruments Co., Bangkok, Thailand) were mixed and dissolved in distilled water at room temperature. Combinations of wall materials at five different mass fractions were studied: *m*(MD)/*m*(GA): 0:100, 25:75, 50:50, 75:25 and 100:0%. Wall material solutions were prepared at 40% (*m*/*m*) solid and kept at 4 °C for 24 h to complete hydration. Solutions of concentrated C-phycocyanin extracted from *Arthrospira* and the wall materials were mixed in a mass ratio of 1:3 (C-PC/wall material). C-PC concentrate without the wall material was used as a control (free C-PC). The solutions were mixed with a high-speed homogeniser (Ultra Turrax, Ika Labortechnik, Staufen, Germany) at 12 000 rpm for 3 min with temperature kept at not higher than 25 °C by cool water in an outer jacket. The mixture was then frozen at -20 °C for 24 h, followed by freeze-drying in a pilot-scale freeze drier (VFD-12SH; Grisrianthon Co., Samutsakorn, Thailand) at pressure ranging 30-60 Pa for 20 h. The dried samples were ground using a mortar and pestle and the powders were packed in polyethylene bags and stored in the dark until required for further analysis. All experiments were performed in triplicate.

### C-phycocyanin concentration

Absorbance of C-phycocyanin was measured at 615 and 652 nm using a UV-visible spectrophotometer (SP-8001; Metertech Inc., Taipei, Taiwan). C-PC concentration (mg/mL) was calculated with the following equation:


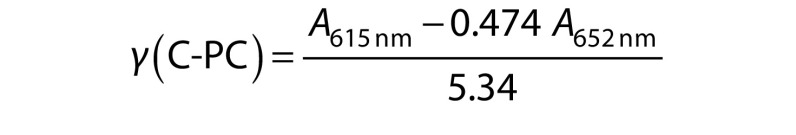


where *A*_615 nm_ is the absorbance of the sample at 615 nm, *A*_652 nm_ is the absorbance at 652 nm, 0.474 and 5.34 are the molar absorption coefficients of C-PC concentration (*28*).

### Determination of microencapsulation efficiency

To evaluate the effectiveness of C-PC microencapsulation, concentrations of C-PC and surface C-PC (SC-PC) of the microcapsules were determined following the modified method of Laokuldilok and Kanha (*29*). For the determination of C-PC, the samples were reconstituted by adding 10 mL distilled water and continuously vibrating on a vortex mixer for 3 min. Then, the mixture was centrifuged at 22 000×*g* and 25 °C for 10 min (Sorvall RC6 Plus Superspeed Centrifuge, Thermo Fisher Scientific Inc., Thermo Scientific). The clear supernatant was collected and filtered through a 0.45-mm pore size Millipore membrane to measure C-PC concentration.

To determine SC-PC concentration, 100 mg of samples were directly extracted with 10 mL of 95% (*V*/*V*) ethanol solution. The mixture was continuously vibrated on a vortex for 30 min, followed by centrifugation at 10 000 rpm and 25 °C for 10 min. After phase separation, the clear supernatant was collected and filtered through a 0.45-mm pore size Millipore membrane, and SC-PC concentration was determined by measuring its absorbance. Microencapsulation efficiency was calculated by the following equation (*26*):


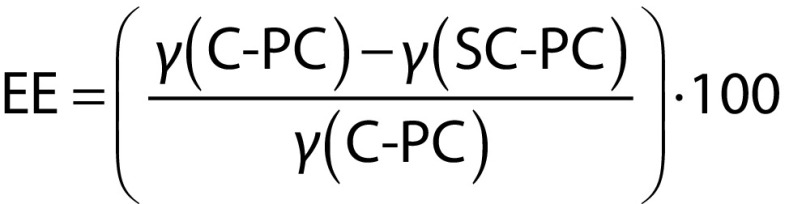


where *γ*(C-PC) is the C-phycocyanin concentration calculated using Eq. 1, and *γ*(SC-PC) is the surface C-phycocyanin concentration.

### Moisture content and water activity

Moisture content of the C-PC microcapsule powder was determined gravimetrically. Samples and aluminium cans were pre-weighed and dried in an oven at 105 °C for 24 h until constant mass. Dry mass of the samples was measured and moisture content was calculated and expressed in percentage.

Water activity (*a*_w_) was measured using the principle resistive electrolytic humidity measuring system at 25 °C (LabMaster-aw, Novasina AG, Lachen, Switzerland).

### Solubility

C-PC microcapsule powder solubility was evaluated following the method of Yamashita *et al.* (*20*). Briefly, samples were dissolved in distilled water and then stirred at room temperature for 30 min. The suspension was then centrifuged at 11 000×*g* for 5 min (Sorvall RC6 Plus Superspeed Centrifuge, Thermo Fisher Scientific Inc., Thermo Scientific). The aliquot supernatant was transferred to a pre-weighed aluminium can and dried at 105 °C in an oven until constant mass. Dry mass of the soluble solid was measured and solubility of the powder product was calculated in %.

### Hygroscopicity

Hygroscopicity of microencapsulated C-PC powder was determined as the tendency of a product to absorb moisture from the surrounding atmosphere. Samples were stored at 20 °C in desiccators which contained saturated sodium chloride solution at 75% relative humidity and *a*_w_=0.75. The samples were weighed before storage and again after 1 week. The hygroscopicity was calculated in grams of absorbed moisture per 100 g of dry solids (*30*).

### Bulk density

A mass of 10 g of C-PC microcapsules was poured into a 10-mL graduated cylinder. Bulk density was calculated by dividing the powder mass by its volume in the cylinder (g/cm^3^) (*31*).

### Colour measurement

Colour of the microencapsulated C-PC powder was measured using a Datacolour Spectraflash Spectrophotometer (SF 600 plus; Datacolour International Co., Lawrenceville, NJ, USA). Colour measurements were expressed in terms of lightness (L*) from 0 (black) to 100 (white) with chromaticity parameters a* from green (-) to red (+) and b* from blue (-) to yellow (+).

### Particle morphology and size distribution

Particle microstructure of C-PC freeze-dried powders was evaluated using a scanning electron microscope (SEM model SU8020; Hitachi High-Technologies Corporation, Tokyo, Japan). Samples were placed in a carbon support and coated with a layer of platinum. The SEM was operated using an acceleration voltage of 5 kV with 5000× and 1000× magnifications. Particle size was measured using a laser light diffraction instrument (Mastersizer 2000, Malvern Panalytical Ltd., Malvern, UK). A small quantity of C-PC microcapsule powder was suspended in isopropanol under magnetic agitation using a sample dispersion unit connected to the equipment. Particle size distribution was observed until successive readings became constant and expressed as D [4,3], the De Brouckere mean diameter was used to characterise a particle (*20*).

### Determination of thermal stability

Thermal stability of C-phycocyanin microencapsulated powders was evaluated using a differential scanning calorimetry (DSC) and thermogravimetric analysis (TGA). In both analyses, a small sample of around 4-6 mg was loaded in a silver pan and crucible for DSC and TGA respectively. An empty pan and crucible were used as reference material. For DSC analyses (DSC3; STAR^e^ system, Mettler Toledo, Greifensee, Switzerland), the pans were sealed and scans were run at a heating rate of 10 °C/min, under nitrogen flow at 50 mL/min from 15 to 250 °C (*32*). Dynamic assays of TGA were performed using a thermobalance (TGA/DSA3+; STAR^e^ system, Mettler Toledo). Temperature programmes for the assays were from 25 to 800 °C at a heating rate of 10 °C/min under nitrogen flow at 50 mL/min (*33*).

### Determination of antioxidant activity

Radical scavenging activity of different wall materials of microencapsulated C-PC powders was evaluated using 2,2-diphenyl-1-picrylhydrazyl (DPPH) antioxidant assay. The samples were dissolved in distilled water. A volume of 2 mL of sample solutions was mixed with 1 mL of 200 µM DPPH in an ethanol solution. The mixtures were incubated at room temperature for 30 min. Absorbance of the mixture was measured at 517 nm by a UV-Vis spectrophotometer (SP-8001; Metertech Inc.). Inhibition (in %) was calculated by the following equation (*34*):


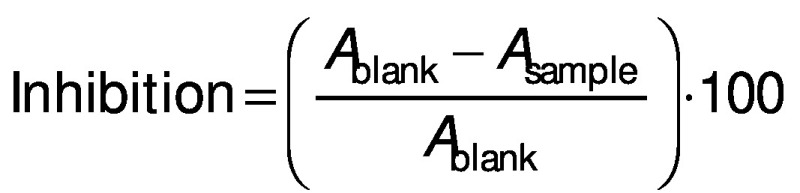


where *A*_blank_ is the absorbance of the control and *A*_sample_ is the absorbance of the sample.

### Statistical analysis

Data were analysed by analysis of variance (ANOVA) using SPSS v. 11.0 (*35*). Duncan’s multiple range test was used to assess significant differences between the samples at p<0.05. All experiments were performed in triplicate.

## RESULTS AND DISCUSSION

### Effect of wall materials on C-phycocyanin microcapsules

Microencapsulation is an enduring technology for protection and controlled release of food ingredients (*36*). For microencapsulated loading of one or more bioactive ingredients, the key functional properties include encapsulation efficiency, size, morphology and also stability during storage (*37*). C-phycocyanin (C-PC), the blue colour from *Arthrospira* sp. is a natural resource, generally recognised as safe (GRAS) for human consumption (*38*). C-PC was extracted from *Arthrospira platensis*, followed by evaporation to increase total solid mass fraction from (1.25±0.26) % (*m*/*m*) in the aqueous extract to (5.20±0.23) % (*m*/*m*) in C-PC concentrate. C-PC concentration in the extract solution was (7.31±0.76) mg/mL, which increased to (18.89±1.11) mg/mL after concentration by evaporation with the volume reduced to a third of the original amount.

Table 1 shows the C-PC mass fractions in the microencapsulated powders and the efficiency of encapsulation after freeze-drying at different fractions of maltodextrin (MD) and gum Arabic (GA) C-PC mass fraction in microcapsules ranged 18.85-20.48 mg/g. A higher fraction of GA in wall material retained a higher C-PC mass fraction in the microcapsules. Moreover, adding GA as the wall material showed better results than using MD for encapsulation efficiency. Our results showed high encapsulation efficiency of freeze-dried C-PC powders. Most commonly used wall materials are maltodextrin, gum Arabic, emulsifying starch and whey protein (*36*). Moreover, modified starch and gelatin were used as wall materials in freeze-drying of turmeric microcapsules (*39*). Ezhilarasi *et al*. (*36*) found that wall materials of whey protein and maltodextrin had excellent encapsulation efficiency during freeze-drying of *Garcinia* fruit extract. Higher encapsulation efficiency of 78-97% was obtained with freeze-drying of *Averrhoa carambola* extract than with spray-drying (*40*).

**Table 1:** C-phycocyanin (C-PC) mass fraction in microencapsulated powders and encapsulation efficiency (EE) using different wall materials

Wall material	*w*(C-phycocyanin)/(mg/g)	EE/%
*m*(maltodextrin/*m*(gum Arabic)
100:0	(18.98±0.65)^a^	(99.75±0.14)^b^
75:25	(18.85±0.37)^a^	(99.95±0.00)^a^
50:50	(19.11±0.32)^a^	(99.92±0.05)^a^
25:75	(20.23±0.64)^b^	(99.94±0.10)^a^
0:100	(20.48±0.27)^b^	(99.90±0.06)^a^

Values are expressed as mean±standard deviation. Different letters indicate significant differences between treatments (p˂0.05)

### Physical properties

Table 2 shows moisture content, water activity (*a*_w_), solubility, hygroscopicity and bulk density of microencapsulated C-PC powders after freeze-drying. Results showed decreasing moisture content with increasing fraction of GA in the wall materials. Moisture content was significantly lowest at 0.99% with 100% GA as the wall material. Moreover, *a*_w_ values were not significantly different when using higher MD, and *a*_w_ decreased with increasing fraction of GA. However, moisture content and *a*_w_ recorded in free C-PC as the control were higher than in C-PC microcapsules. Moisture content and water activity ranged at 0.99-3.38% and 0.07-0.19 respectively for different wall material fractions. Moisture content of food affects its storage, packaging and processing (*20*), while water activity plays a major role in determining both quality change and microbial growth or survival as it indicates the amount of free water available for microbial growth and quality change. To prevent microbial growth, water activity below about 0.6 is needed (*41*). The higher the *a*_w_, the more free water is available for biochemical reactions and shorter shelf life is predicted (*20*). Average *a*_w_ in different wall materials was lower than the *a*_w_ in free C-PC. Therefore, microencapsulated C-PC powders were considered relatively more stable against microbial growth and hydrolytic and enzymatic reactions with *a*_w_ values less than 0.6 (*42*). Moreover, freeze-drying of aqueous lemon by-product extract using maltodextrin and soybean protein formed microparticles with lower moisture content and water activity than those produced by spray-drying (*43*).

**Table 2:** Physical properties of different wall material used for the production of microencapsulated C-phycocyanin (C-PC) powders

Wall material	*w*(moisture)/%	*a*_w_	Solubility/%	Hygroscopicity/%	*ρ_b_*/(g/cm^3^)
*m*(maltodextrin/*m*(gum Arabic)
100:0	(2.4±0.7)^cd^	(0.15±0.00)^b^	(97.1±3.1)^c^	(8.1±0.6)^a^	(0.68±0.05)^c^
75:25	(2.2±0.6)^bc^	(0.18±0.02)^b^	(95.0±0.6)^bc^	(8.5±0.6)^a^	(0.68±0.03)^c^
50:50	(3.4±0.2)^d^	(0.19±0.01)^b^	(93.3±0.4)^b^	(8.9±0.8)^a^	(0.64±0.02)^ab^
25:75	(1.2±0.9)^ab^	(0.08±0.04)^a^	(94.0±1.5)^bc^	(12.8±1.4)^c^	(0.66±0.02)^bc^
0:100	(0.99±0.98)^a^	(0.07±0.01)^a^	(94.8±0.8)^bc^	(13.7±1.05)^c^	(0.68±0.02)^c^
Free C-PC	(7.9±0.3)^e^	(0.26±0.01)^c^	(87.3±4.6)^a^	(11.1±0.3)^b^	(0.62±0.02)^a^

Values are expressed as mean±standard deviation. Different letters indicate significant difference between treatments (p˂0.05)

Solubility, hygroscopicity and bulk density of the different wall materials used in the microencapsulation process are shown in Table 2. Results indicated that all microencapsulated powders had excellent solubility with values ranging from 93.3 to 97.1% and higher than free C-PC. The highest solubility was obtained from C-PC microcapsules with 100% MD wall material. Colourant powders used as ingredients for the food industry must exhibit good solubility. Our results showed that different fractions of maltodextrin and gum Arabic used as wall materials did not affect the solubility values. Hygroscopicity ranged from 8.1 to 13.7%. Higher MD or equal to GA showed lower hygroscopicity, but without significant differences (p˃0.05). Low hygroscopicity of powders resulted in lower adsorption and thus lower molecular mobility (*44*), whereas higher GA fractions in wall materials showed significantly higher hygroscopicity (p˂0.05).

Bulk density of all microencapsulated C-PC powders and free C-PC was around 0.6 g/cm^3^. Wall material composition showed no influence on bulk density. Bulk density of fat powder capsules containing fat and PUFA-rich oil using different wall materials and liquid oil showed no effect on the bulk density and supported this result (*45*). However, bulk properties of food powder are highly dependent on particle size and its distribution (*46*). Moreover, bulk density decreased with the increase in inlet air temperature during encapsulation of vegetable oil by spray drying. Gum Arabic is the most commonly used wall material due to its high soluble fibre content, prebiotic effect, highly digestive tolerance and low caloric value. Gum Arabic is also suitable for various formulations of functional foods as it is non-cariogenic (*47*).

C-PC microcapsules are used as colourants in food products. Colour of microencapsulated C-PC powders and free C-PC revealed that different wall materials had no significant effect on lightness, whereas free C-PC had darker (lower L* value) and deeper blue (b*) colour (Table 3). Blue shade colours (b*) of mixtures of wall materials with high to low fraction of maltodextrin were deeper. Lightness (L*) was lighter in microencapsulated C-PC powders with higher fraction of maltodextrin wall material mixture. Destruction of C-phycocyanin reduced pigment with lighter powders (*40*) that were less blue. Therefore, blue colour of microencapsulated C-PC powders using gum Arabic as wall material gave blue colour (b*) comparable with the control (free C-PC).

**Table 3:** Colour indices and mean diameter particle size of different wall materials used for the production of microencapsulated C-phycocyanin (C-PC) powders

Wall material	Colour			D[4,3]/µm				
*m*(maltodextrin/*m*(gum Arabic)	L*	a*	b*			
100:0	(59.6±1.3)^b^	(-16.4±0.5)^a^	(-24.6±0.5)^a^		(55.2±116)^ab^				
75:25	(59.3±0.8)^b^	(-16.3±0.3)^ab^	(-24.3±0.5)^a^		(5088±0.8)^a^				
50:50	(59.9±3.5)^b^	(-15.2±0.6)^abc^	(-23.2±1.0)^ab^		(54.4±0.7)^ab^				
25:75	(56.2±2.8)^b^	(-14.9±1.4)^bc^	(-21.3±1.9)^b^		(74.3±1.2)^c^				
0:100	(57.3±2.6)^b^	(-14.3±1.0)^c^	(-19.4±1.4)^c^		(72.8±1.2)^c^				
Free C-PC	(40.8±2.6)^a^	(-8.4±1.2)^d^	(-17.5±2.0)^c^		(59.3±5.9)^b^				

Values are expressed as mean±standard deviation. Different letters indicate significant difference between treatments (p˂0.05)

**Fig. 1:**
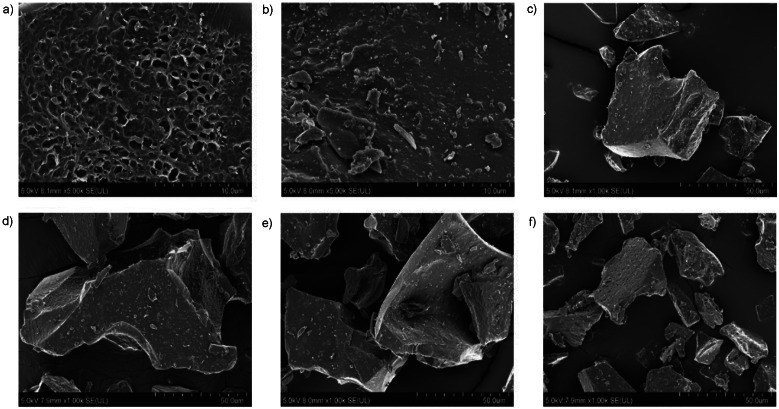
Scanning electron micrographs and particle size distributions of freeze-dried microencapsulated C-phycocyanin (C-PC) powders using different *m*(maltodextrin)/*m*(gum Arabic) as wall materials: a) 100:0, magnification 5000×, b) 75:25, 5000×, c) 50:50, 1000×, d) 25:75, 1000×, e) 0:100, 1000×, and f) free C-PC, 1000×

Fig. 1 shows the external morphology of freeze-dried microencapsulated C-PC powders with different wall materials and particle size distribution with free C-PC as a control. The structure of microparticles was similar to a broken glass pieces of various sizes, which was also found in previous studies (*48*). At low temperatures of freeze-drying the physical state is important for frozen food stability (*49*). A glassy structure with irregular shape might protect the bioactive compounds against heat and oxygen exposure (*20*). The micrographs showed that only 100% maltodextrin used as wall material resulted in porous powders. Loss of porous structure was observed in microcapsules when gum Arabic was added as wall material, possibly due to increased hygroscopicity. However, free C-PC did not have a porous structure.

**Fig. 2:**
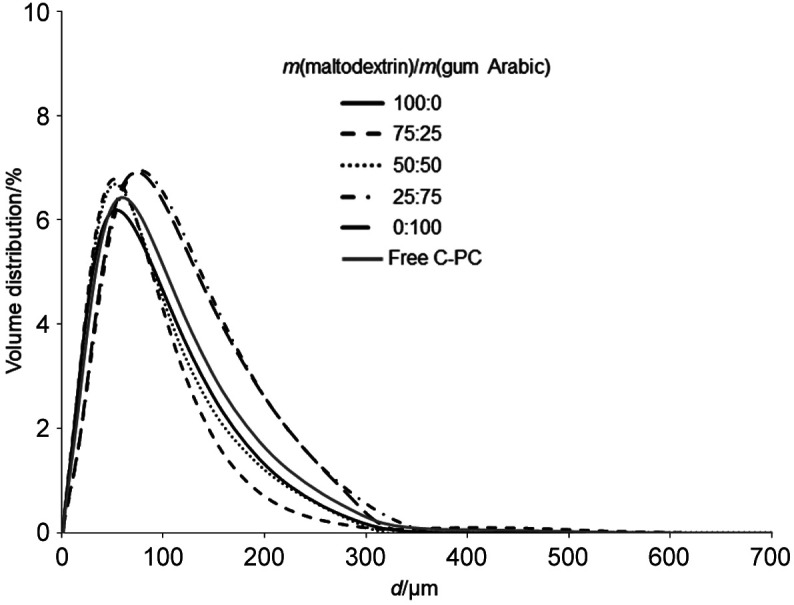
Particle size distribution of freeze-dried microencapsulated C-phycocyanin (C-PC) powders using different fractions of wall materials maltodextrin and gum Arabic

Fig. 2 shows a size distribution graph of all experiments and free C-PC (control) particles. All experiments showed only one distinct peak with particle diameter varying from 1.5 to 316 µm. Mean particle diameters of microencapsulated C-PC powders under different wall material conditions and free C-PC were in the range from 51 to 74 µm (Table 3). Microencapsulated C-PC powder with higher maltodextrin fraction in the wall material mixture had smaller particle size, while higher gum Arabic fraction increased particle size (p˂0.05). Large particle size of freeze-dried samples was caused by the low temperature process and lack of strength necessary to break the frozen drops or to alter the surface during drying. Particle size is related to kinetic solubility which increases as particle size decreases. Moreover, solubility influences particle size dissolution (*50*). Large particle size affects solubility and higher solubility is associated with smaller particle size because of the greater surface area available for hydration (*19*).

### Thermal stability

Table 4 shows DSC and TGA results of the evaluation of C-PC microencapsulated with different fractions of maltodextrin and gum Arabic as wall materials. DSC measures the changes in physical properties of C-PC powder with the change of temperature during time. All experiments show one peak in the DSC thermogram when a homogenous mixture of C-PC and wall materials was used for the production of microencapsulated powder. Glass transition temperature (*t*_g_) was determined as the midpoint of the beginning (*t*_0_) temperature and endpoint (*t*_f_) temperature range of the endothermic peak. Results show that glass transition temperatures of microencapsulated C-PC powders were in the range from 158 to 173 °C, whereas free C-PC had a lower midpoint temperature at 152 °C. Therefore, freeze-dried microencapsulated C-PC powders had higher glass transition temperatures than free C-PC, especially at *m*(maltodextrin)/*m*(gum Arabic)=100:0 and 25:75. Our results showed higher glass transition temperature than freeze-dried blueberry extract with maltodextrin DE 4.0-7.0 at 100.7 °C (*51*). Mass loss from thermogravimetric analysis (TGA) was in the range from 73 to 82% at temperature gradient from 25 to 800 °C. *Arthrospira* cells dried at various temperatures (80-110 °C) had mass loss of 31.7-25.8% at temperature gradients from 180 to 350 °C (*33*). C-PC as a natural pigment extract mixed with other components as wall materials showed high mass loss at high temperatures.

**Table 4:** Thermal analysis of microencapsulated C-phycocyanin (C-PC) powders produced using different wall materials

Wall material	DSC					TGA
*m*(maltodextrin/m(gum Arabic)	*t*_0_/°C	*t*_g_/°C	*t*_f_/°C	∆*H*/(J/g)		Mass loss/%
100:0	169.86	173.00	178.27	-132.76		81.77
75:25	153.71	158.25	173.26	-220.90		76.13
50:50	162.28	164.42	173.19	-188.34		84.92
25:75	169.69	171.42	178.89	-142.39		81.20
0:100	169.42	171.00	176.75	-142.51		77.94
Free C-PC	149.70	152.34	170.96	-170.73		73.20

DSC=differential scanning calorimetry, TGA=thermogravimetric analysis, *t*_0_, *t*_g_ and *t*_f_=initial, glass transition and final temperature respectively, Δ*H*=normalized value

### Antioxidant activity

The antioxidant activity of food is an expression of its capability to defend the human organism from the actions of free radicals and prevent degenerative disorders deriving from persistent oxidative stress. Use of natural antioxidants in the food industry is a promising alternative to synthetic antioxidants and highly compatible for dietary intake with no harmful effects on the human body (*52*). C-phycocyanin has a high antioxidant capacity (*9*) and one of the important characteristics of natural blue C-PC colourants is their scavenging ability for free radicals of reactive oxygen species (ROS). The 50% DPPH free radical scavenging (IC_50_) results from all experiments are presented in Table 5 in the range from 7.6 to 13.5 mg/mL. A higher or equal fraction of maltodextrin to gum Arabic in the wall material mixtures showed lower IC_50_ values, whereas free C-PC had the lowest IC_50_ values with no significant difference. Increasing the gum Arabic fraction of the wall material increased IC_50_ value.

**Table 5:** Antioxidant capacity of microencapsulated C-phycocyanin (C-PC) powders produced using different wall materials

Wall material	*γ*(IC_50_)/(mg/mL)
*m*(maltodextrin/*m*(gum Arabic)
100:0	(8.9±0.2)^ab^
75:25	(8.5±0.3)^ab^
50:50	(8.9±1.1)^ab^
25:75	(10.2±1.3)^b^
0:100	(13.5±1.1)^c^
Free C-PC	(7.6±0.1)^a^

Values are expressed as mean±standard deviation. Different letters indicate significant difference between treatments (p˂0.05), *γ*(IC_50_)=concentration of antioxidant (C-phycocyanin) that causes 50% inhibition of DPPH

## CONCLUSIONS

Selection of suitable wall materials is crucial for the microencapsulation and freeze-drying of C-phycocyanin (C-PC). Wall materials prevent changes due to chemical interaction and maximise retention of the C-PC blue colourant after the drying is completed. A mixture of maltodextrin and gum Arabic was optimised at fraction 25:75, which provided the best conditions for C-PC microencapsulation by freeze-drying. Findings indicated that freeze-dried C-PC microcapsules using a combination of maltodextrin and gum Arabic as wall material offer an interesting alternative to maintaining C-PC colourant stability during encapsulation to produce a powder with high levels of antioxidant blue colourant.
